# Supplementary material for: Peripheral endothelial function can be improved by daily consumption of water containing over 7 ppm of dissolved hydrogen: A randomized controlled trial
Source: PLoS One. 2020 May 29;15(5):e0233484. doi: 10.1371/journal.pone.0233484 (PMC7259729; doi:10.1371/journal.pone.0233484)
Supplement: S1 File — (DOCX) [file pone.0233484.s002.docx]

**Research Protocol**

Project summary

Previous research has demonstrated the benefits of measuring reactive hyperaemia index (RHI) by peripheral arterial tonometry (PAT) in order to assess endothelial function of the vasculature and predict the prognosis of cardiovascular diseases. It is important to identify and examine the factors to improve the RHI. This project aims to estimate the efficacy of molecular hydrogen (H_2_) to improve endothelial function in the peripheral vasculature. In order to do this, we plan to investigate the effect of water (containing a high concentration of H_2_) upon the vasculature of the finger by measuring RHI. Sixty-eight volunteer subjects will be randomly divided into two groups: a placebo group (n=34), who drink water containing molecular nitrogen (N_2_), and a high H_2_ group (n=34), who drink water containing a high concentration of H_2_ (7 ppm H_2_: 3.5 mg H_2_ in 500 mL water). For each group (placebo or high H_2_), RHI is measured prior to taking an initial drink, 1 h and 24 h after the first drink, and 14 days after drinking the water every day. Our hypothesis is that by consuming high H_2_ water, there will be an improvement in RHI.

Protocol title: **Double blinded and placebo controlled examination to observe the effects of the water containing 7ppm molecular hydrogen on the EndPAT test.**

The principal and corresponding investigator and sponsor/funder

Principal investigator: Toru Ishibashi

Huis Ten Bosch Satellite H2 Clinic Hakata, 2-1 Gion, Hakata-ku, Fukuoka 812-0038, Japan.

Anicom Speciality Medical Institute, Sumitomofudosan-Nishishinnjuku-Grand tower 39F, 8-17-1 Nishishinjuku, Shinjuku-ku, Tokyo 161-0023, Japan.

Telephone: +81-92-282-5005

Rationale and background information

Endothelial dysfunction causes the accumulation of vascular damage and induces chronic inflammation which can subsequently lead to atherosclerosis and cardiovascular disease. To estimate the associated risk for diseases related to vascular dysfunction, a range of non-invasive methodologies have been developed to help assess endothelial function. One such method is flow-mediated dilation (FMD) which can monitor the response of the endothelium in conduit arteries. Another method is reactive hyperaemia-peripheral arterial tonometry (RH-PAT) of the small arteries in the fingers. Both of these methods measure vascular hyperaemia response after the brachial artery shuts down and during the subsequent increase of blood flow created by reperfusion. Consequently, these methods are believed to accurately predict risk for cardiovascular events including angina, myocardial infarction, ischaemic stroke, cerebral infarction, acute coronary syndrome and heart failure. However, it is important to identify factors or new methodologies which could improve endothelial function, and prevent endothelial dysfunction. For example, this could be achieved by combining measurements taken independently by both FMD and RH-PAT in order to derive a new prognostic indicator for therapeutic potential. This strategy may also help prevent deleterious events.

Over recent years, research has demonstrated the safety and efficacy of ingesting molecular hydrogen as an anti-inflammatory and anti-oxidant agent^1,2^. We recently demonstrated the safety profile associated with the consumption of water containing over 3.5 mg of dissolved hydrogen and the potential of high H_2_ water to improve FMD methodology^3,4^. While this preliminary finding might explain the nature of the rosy faces often observed in those you regularly consume high H_2_ water, the influence of H_2_ upon resistant arteries, such as the peripheral arteries, which seem to directly reflect the microcirculations of the skin, is still lacking. Direct assessment of the digital artery will provide us with additional information relating to the efficacy of H_2_ in the circulatory system. Because the mechanisms responsible for the relaxation of smooth muscle in a large conduit artery and of small or resistant arteries are different, it is not clear whether the effect of H_2_ upon increased flow from a proximal artery could be transmitted to the distal end, or whether an independent signal initiated by H_2_ could independently stimulate endothelium derived hyperpolarization factor (EDHF) of the microcirculation.

In the present study, we plan to investigate the influence of H_2_ upon reactive hyperaemia of peripheral artery tonometry (RH-PAT) and the effect of daily high H_2_ water consumption upon vasomotor function.

Study goals and objectives

We aimed to investigate the efficacy of high H_2_ water upon endothelial function by measuring the RHI of the finger vasculature after the consumption of high H_2_ water (7 ppm H_2_: 3.5 mg H_2_ in 500 mL water).

Study design and methodology

This study was designed to investigate the possibility of H_2_ acting as an agonist for nitric oxide (NO) and/or EDHF, as suggested previously using FMD^4^. Our study was approved by the Ethics Committee of the Huis Ten Bosch Satellite H2 Clinic. The study population size was determined in accordance with a previous study which demonstrated statistically significant improvement of endothelial function in 34 subjects following the application of high H_2_ water, as assessed by FMD^4^. For the present study, we aimed for a population size of 68 patients, double that of the previous study. All participants are informed individually about the significance of the study and the instruments to be used for measurements. Participants are formally recruited into the study after providing their written informed consent. It is important that none of the participants had received medications, medical treatments or dietary supplements for at least 6 months prior to our study. Once recruited, the participants are randomly divided into two groups: a high H_2_ group (who drink high H_2_ water) and a placebo group (who drink placebo water). All participants are asked to fast, and avoid drinking caffeinated drinks or ingesting sugar, for 6 hours before testing.

In accordance with previous studies^5-7^, RHI is measured using endothelium-dependent digital pulse amplitude testing (EndoPAT), with the aid of an EndPad2000 System (Itamar Medical Inc.). In brief, disposable RH-PAT probes are placed individually on both 2d fingers and a blood pressure cuff is placed on the subject’s ipsilateral upper arm region in a quiet and dimmed room. After resting for more than 20 min, baseline pulse amplitude recording is initiated and RH-PAT induced via reperfusion of blood flow after a 5-min cuff occlusion of the brachial artery at 60 mmHg above the measured systolic pressure. Next, the participants drink 500 ml of placebo or high H_2_ water within 10 min, and after resting for 1 h, the RHI is measured again. On the second day, 24 h after the first ingestion of placebo or high H_2_ water, and before the second ingestion, the RHI is re-measured. After the measurements are obtained, participants take a second drink, and the day after, they drink the placebo or high H_2_ water once each day until the day before the last measurement (day 14). In total, the participants drink the high H_2_ water, or placebo, on 14 different occasions.

The high H_2_ water (Hydrogen water 7.0; Ecomo International Co., Ltd., Iizuka-shi, Fukuoka, Japan) is prepared according to a method described previously^3^. In brief, hydrogen gas is produced in an elastic polyethylene terephthalate (PET) bottle manufactured by TOMIKAWA Chemical Industry Co., LTD. The bottle is then filled with 530 ml of water by mixing 75% of metal aluminium grains with 25% of calcium hydroxide (by weight) and 0.5 ml water in an acrylic resin tube placed into the bottle. This reaction causes hydrogen gas to be dissolved in the water. The placebo water is prepared by filling up a PET bottle containing water with molecular nitrogen (N_2_) gas under 0.8 MPa to make the placebo bottles as firm as the bottles containing the high H_2_ water. An acrylic resin tube with a placebo non-woven fabric (used to produce H_2_ gas in a tube but lacking the reactive compound) is also placed in the placebo bottles so that the volunteers cannot distinguish between the two types of water.

Safety considerations and follow-up

Molecular hydrogen is a stable molecule, requires 436 kJ/mol of energy to cleave its covalent bonds and reacts only with radical molecules of extremely high reactivity such as hydroxyl radical and peroxynitrite, both of which are virulent in human cells. Furthermore, the ingestion of molecular hydrogen, as an anti-inflammatory and anti-oxidant agent, has been established as being safe^1-4^. Nevertheless, when volunteers complain of any unusual physical conditions, the participants immediately cease assessment and are treated appropriately. All participants are then followed-up for at least two months, but are welcome to approach us thereafter if they are concerned about unusual physical conditions and if they have any further queries related to this research.

Data management and statistical analysis

The efficacy of high H_2_ water was estimated by analysing the change in RHI at each time point based on the value of RHI before drinking, thus reflecting changes in RHI from baseline. Using data acquired at 1 h, 24 h, and 2 week time-points, we created a mixed effects model for repeated measures (MMRM) including the treatment group, time points, and the interaction between treatment group and time points as fixed effects, with the RHI at baseline as a covariate. The MMRM does not assume a specific covariance structure for error variance. Under the MMRM, the adjusted mean change from baseline, the adjusted mean difference between treatment groups, and their 95% confidence intervals (CIs) and *p* values, were calculated using the SAS software, version 9.2 (SAS Institute, Inc. Cary, NC, USA).

Ethics

This study was designed to assess whether H_2_ can act as an agonist for NO and/or EDHF, as indicated by our previous research using FMD. The research protocol was registered with the UMIN clinical trial registry (number: UMIN000032510; dated 08/05/2018). Our study was approved by the Ethics Committee of Huis Ten Bosch Satellite H2 Clinic following discussion relating to the safety and significance of this study. After enrolment and the provision of informed written consent, all volunteers are informed individually about the significance of the study, the properties and safety of molecular hydrogen, the high H_2_ water and the placebo water, and the instruments of intended measurement.

**References**

1. Ichihara, M. *et al.* Beneficial biological effects and the underlying mechanisms of molecular hydrogen - comprehensive review of 321 original articles. *Med Gas Res.* **5**, 12 (2015).

2. Ishibashi, T. Molecular hydrogen: new antioxidant and anti-inflammatory therapy for rheumatoid arthritis and related diseases. *Curr Pharm Des.* **19**, 6375–6381 (2013).

3. Ishibashi, T. *et al.* Consumption of water containing a high concentration of molecular hydrogen reduces oxidative stress and disease activity in patients with rheumatoid arthritis: an open-label pilot study. *Med Gas Res.* **2**, 27 (2012).

4. Sakai, T. *et al.* Consumption of water containing over 3.5 mg of dissolved hydrogen could improve vascular endothelial function. *Vasc Health Risk Manag.* **10**, 591–597 (2014).

5. Matsuzawa, Y. *et al.* Peripheral endothelial function and cardiovascular events in high-risk patients. *J Am Heart Assoc.* **2**, e000426 (2013).

6. Bonetti, P. O. *et al.* Enhanced external counterpulsation improves endothelial function in patients with symptomatic coronary artery disease. *J Am Coll Cardiol.* **41**, 1761–1768 (2003).

7. Matsue, Y. *et al.* Peripheral microvascular dysfunction predicts residual risk in coronary artery disease patients on statin therapy. *Atherosclerosis.* **232**, 186–190 (2014).

Informed Consent Forms

-------------------------------------------------------------------------------------------------------------------

Informed Consent form for men and women who participate in our research project entitled “Double-blinded and placebo-controlled examination to observe the effects of the water containing 7ppm molecular hydrogen.”

Information about this study

Molecular hydrogen (H_2_) is a fundamental and safe gas molecule. Because H_2_ is stable, it reacts only with dangerous radical molecules in our body; it is not thought to react with the components of our body including protein, DNA and other essential molecules. Previous research has shown that high H_2_ water has anti-inflammatory effects against chronic inflammation including rheumatoid arthritis by scavenging dangerous radicals including hydroxyl radical.

Purpose of the study

In our previous study, we established, using the FMD test, that endothelial function was improved by treatment with high H_2_ water containing including 7ppm H_2_. Here, using EndoPAT, we aim to investigate the effects of H_2_ water on the endothelium of peripheral arteries. We expect that the effects of H_2_ water can lead to an improvement in endothelial function which is vital for a healthy life and the avoidance of cardiovascular disorders.

You are a healthy person and have not taken ant medications or dietary supplements, or received any medical treatments, for at least 6 months prior to this study. You are asked to fast, and avoid drinking caffeinated drinks or ingesting sugar for 6 hours before the test. Your participation is voluntary and you can choose to participate or not.

You will be randomly divided into one of two groups: either a high H_2_ group (who drink high H_2_ water) or a placebo group (who drink placebo water). During this study, you will therefore drink high H_2_ water (containing 7 ppm H_2_: 3.5mg H_2_ in 500 mL water) or placebo water (containing N_2_ in 500 mL water) once every day for 14 days. The EndPAT test will be performed 4 times, before you drink high H_2_ or placebo water, 1h after your 1^st^ drink, on the second day, and the day after you have drunk the water 14 times. No side effects have been reported with high H_2_ water or the water containing N_2_. You will be followed-up for at least two months after this study and can approach us any time thereafter if you experience unusual physical conditions.

Confidentiality

The information that we collect from this study will be kept confidential. No one but the researchers overseeing this project will be able to see this information. Personal information or data that can identify you will never be published. The data obtained in this study will only be used for medical research and never used for any other purpose. If you wish, you can request the data collected in this study after the research has finished. You can also stop participating in this research whenever you wish.

------------------------------------------------------------------------------------------------------------------

I have read all of the foregoing information, have been given a full explanation of this study and understand what is required by participation in this project. All questions have been answered to my satisfaction. I consent voluntarily to participate in this clinical research.

Print Name of Participant:

Signature:

Date (year/month/day)

Principal investigator

Toru Ishibashi

Huis Ten Bosch Satellite H2 Clinic Hakata, 2-1 Gion, Hakata-ku, Fukuoka 812-0038, Japan

Anicom Speciality Medical Institute, Sumitomofudosan-Nishishinnjuku-Grand tower 39F, 8-17-1 Nishishinjuku, Shinjuku-ku, Tokyo 161-0023, Japan.
