# Supplementary material for: Peripheral endothelial function can be improved by daily consumption of water containing over 7 ppm of dissolved hydrogen: A randomized controlled trial
Source: PLoS One. 2020 May 29;15(5):e0233484. doi: 10.1371/journal.pone.0233484 (PMC7259729; doi:10.1371/journal.pone.0233484)
Supplement: S2 File — (DOCX) [file pone.0233484.s003.docx]

**研究プロトコール**

タイトル：「エンドパットテストにおける7ppm水素水の飲用効果を観察するための二重盲検試験」

【概要】 これまでの研究で末梢動脈の反応性充血に対する指標(RHI)は、心臓血管疾患の予後を予測するのに有用であることが示されています。したがってこのRHIを改善する因子をテストし同定することは重要です。本研究では、水素分子が末梢血管内皮細胞機能を改善できるか評価するために、高濃度水素分子を含む水素水の手指血管への影響を、RHIを測定することによって調べることを目的としています。６８名の被験者は無作為にプラセボグループ（窒素が溶かされた水）と7ppm高濃度水素水を飲むグループの二つに分け(両者とも500ml, 水素含有量は3.5mgとなります)、それぞれのグループで最初に試験用の水を飲む前と、飲んだ後、１時間後と２４時間後、そして毎日飲んで２４時間後にRHIを測定します。私たちは、水素水を摂取することでRHIが改善すると考えています。

検査責任者

石橋　徹

ハウステンボスサテライトH2クリニック博多

〒812-0038 福岡市博多区祇園2-1 Tel: 092-282-5005

アニコム先進医療研究所

〒161-0023東京都新宿区西新宿8-17-1住友不動産西新宿グランドタワー39F

【理論的根拠および研究の背景】

血管は、裏打ち細胞である血管内皮細胞の機能によって収縮したり拡張したりすることによって、血流に応じて血液を流す役割を担っています。この内皮細胞の働きが悪くなると、血管が傷つき、血栓やプラークができやすくなります。その結果、動脈硬化が進行し、ひいては心筋梗塞、脳梗塞といった重篤な病気になる危険性があります。 高濃度の水素分子を含む水を飲用することによって、血管内皮細胞の機能が簡便に改善することが期待されています。血管内皮細胞機能を検査する方法として、上腕動脈の血管内皮血流依存性血管拡張反(FMD) を検査する方法および、手指の小血管の血管内皮機能を調べる指尖動脈反応性充血検査(RH-PAT)の二つがあり、どちらも、上腕動脈を駆血した後再還流させ、検査するものです。これら二つの血管内皮機能検査を組み合わせることによって、心臓血管病変や悪いイベントを予防することができると期待されています。

これまでの研究で、水素の安全性と抗炎症作用、抗酸化作用が示されています^1,2^。また、我々のこれまでの研究で、3.5mgの水素を含む水素水（7ppm）を飲用することで、上記FMDを安全に改善することを明らかされました。これによって、飲用者の顔色が良くなるメカニズムが示唆されましたが、皮膚の微小循環に直接影響する末梢動脈の機能についてのテストはまだなされていませんでした^3,4^。大きな血管と、微小な血管とでは平滑筋による拡張メカニズムが異なると考えられているので、これを調べることで、水素の効果に関し、水素が直接EDHFと呼ばれる微小循環を拡張するシグナルに直接作用するのかどうかなど、さらなる情報が得られると考えられます。

【研究の目的】

7ppmの水素水500mlを飲むことで、RHIを指標とした血管内皮細胞機能への水素分子の影響を明らかにすることを目的としています。本試験は、以前のFMD についての試験結果から示唆された、血管拡張因子であるNOガスあるいはEDHFに対し水素分子がアゴニストとして作用するかどうかを調べるためにデザインされ、試験の被験者数は前回の34名を対象にしたFMD試験^4^の結果をもとに68名にて統計的に有用な情報が得られると考え、規模を決定し、ハウステンボスサテライトH2クリニックの倫理委員会の承認のもと行われます。全ての試験参加者の皆様には、一人一人この研究の意義と、測定に使う機器の説明を事前に行い、書面による同意書の提出をもって参加者といたします。参加を検討される場合、本試験の最低６ヶ月以内に、あらゆる医学的治療やサプリメントの摂取をしていないことが前提です。これら条件を満たして参加が決まった場合、二重盲検試験であるので、プラセボ水を飲用するグループと水素水を飲用するグループのどちらかに、検査する者もわからないように振り分けられます。被験者の皆様は、エンドパット試験の前６時間以内は絶食で、カフェインや糖質を含む水分も摂取してはいけません。

RHIの測定方法ですが、エンドパット2000システムを用います^5-7^。血圧測定の時のように片方の上腕部に駆血帯を巻き、両方の示指に測定装置を装着し、刺激のない静かな暗い部屋で20分静かに安静を保った後、収縮期血圧プラス60mmHgの圧力で５分間駆血しRHIを測定します。試験用の水を飲用する前に最初の測定を行い、その後10分以内に最初の500mlを飲み、その１時間後、２４時間後にそれぞれ、二日目の飲用前に測定します。翌日からは朝決まった時間に1日１本を飲用し、６時間以上食事を取っていない午後の時間帯に一回測定し、合計１４日間の測定となります。

高濃度水素水はエコモ社の水素水7.0^3^を用います。ペットボトルの中で飲用水と触れないようにアルミニウムと水酸化カルシウムを用いて水素ガスを発生させ、内圧を上げることによって7ppmの水素水を作成し、飲用します。プラセボ水は0.8MPaの圧で同じペットボトルの水に窒素ガスを封入したものを用います。両者は見た目上、ボランティアの皆様および試験者に区別がつきません。

【安全性および倫理規程について】

水素ガスは水素イオンと電子に解離させるためには436kcal/molものエネルギーが必要と考えられており、非酵素的に反応できるのは、ヒドロキシラジカルやペルオキシナイトライトなど非常に反応性が高く有害なラジカル分子だけであることから、安全と考えられています^1-4^。これまでの抗炎症、抗酸化作用に関する研究も含め、有害事象は観察されておりません。また、水素ガス自体、腸内細菌も生産しており、私たちヒトの体内に普段から含まれております。このように安全性は確立されていると思われますが、被験者に対しては、検査終了後も２ヶ月間は何か普段と体調が違うと感じられた場合など、あるいはさらに質問がある場合は問い合わせていただくよう説明いたします。データは最初の飲用前のRHI値を基準値とし、SASソフトウェアを用いて各測定値を統計処理することによって測定結果を評価します。この研究は、ハウステンボスサテライトH２クリニックにおける倫理委員会の承認のもと行われます。また試験は、UMIN clinical trial registryに登録されます。 (number: UMIN000032510; dated 08/05/2018). 自発的に協力する方が被験者となり、すべての被験者の方々はこの試験の意義や方法、高濃度水素水およびプラセボ水の詳細および安全性などに関する全ての説明を受け、この研究に参加することによって求められることを理解した上で、同意書に署名し提出した上で参加します。

**References**

1. Ichihara, M. *et al.* Beneficial biological effects and the underlying mechanisms of molecular hydrogen - comprehensive review of 321 original articles. *Med Gas Res.* **5**, 12 (2015).

2. Ishibashi, T. Molecular hydrogen: new antioxidant and anti-inflammatory therapy for rheumatoid arthritis and related diseases. *Curr Pharm Des.* **19**, 6375–6381 (2013).

3. Ishibashi, T. *et al.* Consumption of water containing a high concentration of molecular hydrogen reduces oxidative stress and disease activity in patients with rheumatoid arthritis: an open-label pilot study. *Med Gas Res.* **2**, 27 (2012).

4. Sakai, T. *et al.* Consumption of water containing over 3.5 mg of dissolved hydrogen could improve vascular endothelial function. *Vasc Health Risk Manag.* **10**, 591–597 (2014).

5. Matsuzawa, Y. *et al.* Peripheral endothelial function and cardiovascular events in high-risk patients. *J Am Heart Assoc.* **2**, e000426 (2013).

6. Bonetti, P. O. *et al.* Enhanced external counterpulsation improves endothelial function in patients with symptomatic coronary artery disease. *J Am Coll Cardiol.* **41**, 1761–1768 (2003).

7. Matsue, Y. *et al.* Peripheral microvascular dysfunction predicts residual risk in coronary artery disease patients on statin therapy. *Atherosclerosis.* **232**, 186–190 (2014).

「エンドパットテストにおける7ppm水素水の飲用効果を観察するための二重盲検試験」についての説明・同意書文書

この同意書は臨床研究の目的と内容を説明し、協力への同意を確認するものです。

【研究の意義、目的】

水素分子は基本的に安定なため、生体内のタンパク質やDNA、その他の重要な分子と反応せず、非常に反応性が高く危険なラジカル分子とのみ反応すると考えられています。これまでの研究で、高濃度水素水は関節リウマチなどを含む慢性炎症に対し、ヒドロキシラジカルなど危険なラジカル分子を打ち消すことによって抗炎症作用を持つことが示されています。

これまでの研究において7ppmの水素水がFMDテストを用いた血管内皮検査にて内皮機能が改善されることが示されています。今回は、エンドパットテストを用いて、末梢動脈への水素分子の影響を調べます。我々は水素水が健康維持に寄与し、心臓血管異常にならないように血管内皮機能を改善することを期待しています。

【参加要件】

あなたは健康であり、最近６ヶ月以内に医学的治療受けておらず、サプリメントの摂取をしていないとこを確認します。このテストを受ける６時間以内は絶食で、カフェインや糖質を摂ってはいけません。

上記要件を満たして参加が決まった場合、二重盲検試験であるので、プラセボ水を飲用するグループと水素水を飲用するグループのどちらかに、検査する者にわからないように振り分けられます。血圧測定の時のように片方の上腕部に駆血帯を巻き、両方の示指に測定装置を装着し、刺激のない静かな暗い部屋で20分静かに安静を保った後、収縮期血圧プラス60mmHgの圧力で５分間駆血しRHIを測定します。試験用の水を最初に飲用する前に測定を行い、その後10分以内に最初の500mlを飲み、その１時間後、２４時間後と、二日目の飲用前に測定します。その翌日からは朝決まった時間に1日１本を飲用し、その後６時間以上食事を取っていない午後の時間帯に一回測定し、合計１４日間の測定となります。

【守秘義務】

この試験で得られた情報は守秘されます。本試験に携わる研究者以外が被験者の情報を見ることはできません。被験者個人が特定されるようなデータや情報は決して公開されません。希望される場合は、この試験終了後にデータを要求することができます。あなたは、試験の期間中、いつでも本試験を中止することができます。

------------------------------------------------------------------------------------------------------------------

私はこの試験に関する全ての説明を受け、この研究に参加することによって求められることを理解しました。私の質問は、すべて満足するまで答えていただきました。私は自発的にこの臨床研究に参加することに同意します。

参加者　住所

氏名（署名）:

平成　　年　　月　　日

研究責任者

石橋　徹

ハウステンボスサテライトH2クリニック博多

〒812-0038 福岡市博多区祇園2-1

アニコム先進医療研究所

〒161-0023東京都新宿区西新宿8-17-1住友不動産西新宿グランドタワー39F
