# Supplementary material for: Peripheral endothelial function can be improved by daily consumption of water containing over 7 ppm of dissolved hydrogen: A randomized controlled trial
Source: PLoS One. 2020 May 29;15(5):e0233484. doi: 10.1371/journal.pone.0233484 (PMC7259729; doi:10.1371/journal.pone.0233484)
Supplement: Attachment — Submitted filename: Response to reviewers.docx [file pone.0233484.s005.docx]

**Responses to the Reviewers’ Comments**

5. Review Comments to the Author

Please use the space provided to explain your answers to the questions above. You may also include additional comments for the author, including concerns about dual publication, research ethics, or publication ethics. (Please upload your review as an attachment if it exceeds 20,000 characters)

Reviewer #1: In this manuscript, Ishibashi et al. describe the effects of hydrogen water for 2 weeks on endothelial function assessed by reactive hyperemic index (RHI) in 68 Japanese volunteers. The authors observed that hydrogen water significantly increased RHI in these subjects. The topic that the authors investigated is clinically important. However, there are critical points that the authors should address.

Comments
**Comment 1:** The specific purpose of the study is vaguely stated. In addition, conclusion is also vaguely stated. The present study lacks a real atherosclerotic group with endothelial function (e.g., hypertensive patients, diabetes mellitus, dyslipidemia, and cardiovascular disease).

**Response:** Thank you very much for your suggestions.

The purposes of the present study were to assess the effects of H_2_ on endothelial function before morbidity and to provide the primary data of healthy people before applying H_2_ to a group with diseases related to endothelial dysfunction including atherosclerosis. Our study aims have been clearly stated in the Abstract section (page 2, lines 23-24) and Introduction section (page 5, lines 63-67; page 6, lines 79-83). The EndPad examination itself is the method used to prevent atherosclerosis before the subjects are involved in the group that already developed the diseases.

Therefore, the aims of the study were to investigate the effects of H_2_ on the endothelium of healthy populations and to determine whether H_2_ has beneficial effects in improving endothelial function before atherosclerosis and cardiovascular diseases develop. This is a basic study focusing on the primary effects of H_2_ on endothelial function of a healthy group, not people with morbidities, as noted in the text.

Page 2, lines 23-24_

“In this study, we aimed to investigate the effect of H_2_ on RH-PAT of the small arteries of fingers in healthy people.”

Page 5, lines 63-67

“It is important to not only estimate the function of the endothelium of both conduit arteries and peripheral arteries in healthy people using these non-invasive methodologies, but to also identify the factors or methodologies that can improve endothelial function and prevent endothelial dysfunction in healthy people without administering pharmaceutical drugs.”

Page 6, lines 76-80

“Besides these disease-conditions, it is important to identify safe and conventional ways for achieving relatively high (healthy) RHIs in the small vessels of healthy individuals.”

Additionally, one of the factors that made the Discussion section of our manuscript too long is the limitation of the insight where the effects of H_2_ is originated only for the scavenger property against hydroxyl radical. Many researchers and clinicians in this field are pointing to the limitations of the scavenger theory. Our study’s result cannot be explained by the diminishment of hydroxyl radicals and/or peroxynitrite by H_2_.

Recently, we proposed new mechanistic insights of H_2_ aside from the scavenger theory, and the first evidence to explain the insights was accepted at the end of 2019 after we submitted this manuscript and published in February 2020 [35]. We have shortened the Discussion and restructured it to make it concise and clear by adding explanations to the mechanisms by which H_2_ could improve the endothelial functions aside from the scavenger theory (page 22, line 311 to page 23, line 334).

**Comment 2:** A number of studies in vitro reported that hydrogen directly works several types of cells through the various mechanisms. However, it is doubt that dissolved hydrogen in hydrogen water is taken in the vasculature and the cell after drinking hydrogen water in humans.

**Response:** It has been clarified and agreed in this field that H_2_ is transferred into the human body and circulated from the ingested water containing H_2_. In cases where H_2_ was dissolved in water (15 ml/kg of 0.8-mM H_2_ water was administered to rats), H_2_ effectively alleviated nephrotoxicity caused by cisplatin at a concentration of 5-6 μM of H_2_ in blood [22]. In a clinical report about the concentration of H_2_ in blood 30 minutes after ingesting water containing H_2_, we also clarified that presence of hydrogen gas during exhalation from the lung of the person who digested the high H_2_ water can be explained by the rapid and passive diffusion of H_2_ in the aqueous human body including circulation [21].

We have added this explanation to the Methods section (page 9, line 140 to page 10, line 148).

[22] Nakashima-Kamimura N, Mori T, Ohsawa I, Asoh S, Ohta S. Molecular hydrogen alleviates nephrotoxicity induced by an anti-cancer drug cisplatin without compromising anti-tumor activity in mice. Cancer Chemother Pharmacol. 2009;64: 753-761.

**Comment 3:** Subjects enrolled in this study were relatively young and healthy subjects. However, values of RHI were markedly small (e.g., 0.61 in the placebo group and 0.63 in the hydrogen water group). In addition, the authors stated that the border value of 0.71 seems to be close to that of the healthy masses (page 25, lines 6 and 7). This interpretation is clearly wrong. Previous studies including guidelines for management of endothelial function have clearly shown that normal range of RHI (healthy endothelial function) is ≥2.10, borderline is 1.67 to 2.10, and abnormal is <1.67.

**Response:** The value you noted is not the natural logarithmic scaled RHI (Ln_RHI). Because RHI values are not normally distributed, RHI here is Ln_RHI, as described in the Methods section, [RHI=Ln{[RH-PAT ratio]×[0.226×Ln (baseline)–0.2]}[2,3, 20]. Therefore, most of the clinical trials using RHI including the large-scale clinicals trials usually adopt Ln_RHI of RHI, and some of them express the Ln_RHI simply as RHI.

We have added “and we used the natural logarithmic scaled Ln_RHI” in the Methods (page 8, lines 115-117) and changed “RHI” to “Ln_RHI” in the Abstract (page 2, lines 27, 30, 34, 35), Methods (page 10, lines 151, 152, 153, 157), Results (page 13, lines 182, 185, 195, 196; page 14, lines 199, 201, 203, 206; page 15, lines 211, 213; page 16, lines 215, 217, 219, 221, 223, 225, 227; page 17, lines 229, 231; page 18, line 234), and Discussion sections (page 18, lines 241, 243, 245, 247, 248, 249; page 19, lines 252, 258, 260, 261; page 20, lines 267, 269, 271; page 22, line 310)

**Comment 4:** Unfortunately, there are no mechanisms by which hydrogen water significantly increased RHI in healthy subjects. As the authors stated in the Discussion section, measurements of oxidative stress and inflammatory markers would draw more specific conclusions concerning the role of dissolved hydrogen in hydrogen water on vascular function.

**Response:** Recently, we proposed the new mechanistic insights of H_2_ aside from the scavenger theory, and the first evidence to explain the insights were accepted at the end of 2019 after we submitted this manuscript. We have shortened the Discussion and restructured it to make it concise and clear by adding the explanations for the mechanisms by which H_2_ can improve endothelial functions aside from the scavenger theory, as follows (page 22, line 311 to page 23, line 334).

“It should be noted that measuring RH of the peripheral microcirculation in which occlusion of the brachial artery following the release of blood flow is known as the model of ischemia reperfusion (I/R) injury, although the function of the vasodilation of the microvasculature is not fully understood [31]. The mechanisms of the cellular damage by I/R injury are believed to be caused by the superoxide burst in the mitochondrial respiratory chain. Particularly, the reverse electron transport accompanied by the elevated mitochondrial membrane potential is the major factor of electron leakage in mitochondrial complex I [32]. Recently, we have reported that H_2_ reduces the mitochondrial membrane potential in the living cultured cells [33]. In the study, the overproduction of superoxide with the high ratio of NADH/NAD^+^, which reflects the highly reduced state of mitochondria during hypoxia following I/R injury [34], was suppressed by H_2_. This situation corresponds to the right circle in Figure 4. On the basis of these most recent mechanistic insights of H_2_ [33, 35], we suppose that H_2_ may improve endothelial function via the mitochondrial mechanisms in healthy subjects. H_2_ may shift the circle in Figure 4 from right to left by reducing the elevated mitochondrial membrane potential as well as the overproduction of superoxide under the condition with hypoxia presented in the right circle.

Thus far, the limitations to explain the mechanisms of the H_2_ effect on a wide range of diseases or pathological states based on the scavenging property of H_2_ with respect to highly reactive radicals have been discussed [36, 37]. In blood flow, the availability of H_2_ and/or the frequency with which it reacts with target radicals seems insufficient to explain the distinct biological effects of H_2_ in the peripheral vasculature. In the present study, the continuous scavenging of deleterious radicals by H_2_ is excluded by the single intake of high H_2_ water in a day. Therefore, we consider that the effects of H_2_ herein are occur via the mitochondrial mechanisms described in Figure 4 [32-35].”

**Comment 5:** It is well known that blood pressure, heart rate, glucose metabolism, and lipid profiles) other than BMI also are confounding factors for endothelial function. These parameters should be presented in Tables.

**Response:** The alternations of blood pressure and heart rates have been added to Table 1.

We have added the comment below as a study limitation at the end of the Discussion section (page 24, lines 336-340).

As for glucose metabolism and lipid profiles, which are confounding factors for endothelial function, we did not collect blood samples, and our study protocol did not include the blood test.

**Comment 6:** Introduction section and the Discussion section are too long. They should be significantly shortened as they contain a number of issues not directly related to the message of this study.

**Response:** Thank you for your suggestion. We have deleted unnecessary sentences and restructured the Introduction section.

Introduction, page 5, line 71 to page 6, line 78

“In some interventional studies, attempts to improve the reactive hyperemia index (RHI) without using a pharmaceutical approach, but instead focusing on exercise therapy and/or lifestyle improvement, have been reported in patients suffering from heart failure and metabolic syndrome [15-18]. Improvements in RHI were also reported when postprandial hyperglycemia was treated pharmaceutically [19]. Besides these disease conditions, it is important to identify safe and conventional ways for achieving relatively high (healthy) RHIs in the small vessels of healthy individuals.”

Deleted sentences

“Although the evidence regarding the use of RH-PAT is lacking, the results obtained and reported after using this relatively new method are similar to those obtained for FMD, and the convenience and/or reproducibility of the methodologies are more universal in RH-PAT compared to that in FMD”

“This type of evaluation can elucidate the prognostic value of a new therapeutic modality, as well as determine the strategies for preventing any deleterious events associated with the use of this method.”

“So far, we have discussed the potential that H_2_ can react and dissociate to donate electrons to the highly active radical molecules such as ^•^OH and ONOO^-^. According to guidelines on the safety of ingesting H_2_, therapeutic applications of H_2_ including clinical approaches have been emerging in the past decade.”

“Apart from the redox-related signalling including NO and the surrounding radical molecules such as H_2_O_2_, EDHF is attributed to electrochemical reactions by hyperpolarization of the endothelial cells first. The changes in the membrane potential of the endothelial cells can reach -85 〜 -80 mv, which is 10 〜 30 mv below the basal membrane potential of arteries and arterioles. This hyperpolarization is dependent on the activation of both endothelial Ca^2+^-sensitive K^+^-channels, the small K_Ca_2.3 and K_Ca_3.1. The efflux of K^+^ affecting the contiguous smooth muscle cells connected to the gap junctions is the primary factor in the mechanism of vasodilation of relatively small arteries. Because H_2_ is a stable molecule requiring 436 kJ/mol to cleave the covalent bond, and this property simultaneously permits the safety of H_2_ in our body, it is unlikely that the chemical reactions of H_2_ are directly involved in the mechanisms of EDHF.”

“In this study, healthy meant that no medications were required and/or not likely to become ill. In this study, however, quite a few subjects had RHI values below 0.71 despite their thinking that they were healthy (22 subjects in the placebo group and 19 subjects in the high H_2_ group).”

“…probably because of the single overload of the volume on the peripheral vasculature in the condition of low RH. The effects of the intake of 500 ml of water within 10 min is thought to have caused the increase in the volume of blood flow and affected the microcirculation independent of the presence of H_2_ or N_2_ in the water for individuals not accustomed to drinking such types of water. The RH-PAT in the small vessels may be influenced by volume loading more than by the diameter of the brachial artery as measured in FMD test. Furthermore, such loading of volume during the test may strain the sympathetic nerve and affect the PAT data. Within the high H_2_ group, an improvement was seen at 1 h after the drink in addition to at 24 h and 2 weeks after ingestion of the drink. This is consistent with the results we previously reported using FMD, where an H_2_-specific improvement was observed within 1 h after the consumption of 3.5 mg of H_2_. Currently, however, we could not assure the immediate efficacy of H_2_ on the peripheral artery, because of the presence of the placebo effect at 1 h in the low RHI group. Regarding the effect of H_2_ on RHI at 24 h, there was significant improvement from the RHI before consumption only within the H_2_ groups (Table 4), whereas in the low RHI groups, the difference from the value before ingestion was improved in the both of the placebo and H_2_ group. Further investigations about the immediate effects of a single ingestion of H_2_ within 24 h are necessary.”

**Comment 7:** Figure 4 is too speculative and is not needed.

**Response:** According to your suggestion, we have revised Figure 4 and deleting the schema below the circles. The remaining circles summarize the previous publications that are necessary for understanding our data here with the mitochondrial membrane potential, which is affected by H_2_, particularly for the researchers working on H_2_ medicine outside of the field of vasculature. We have also revised and shortened the Discussion section with regard to Figure 4, as described above (page 21, line 286 to page 23 line 334).

Reviewer #2: The manuscript addresses an interesting topic. The data are original and rich of information. The statistical methods employed are sound. Results are of potential interest. Some comments follow.

**Comment 1:** The data are not fully available. This is not in line with the journal's guidelines. More importantly, this does not allow the reviewer to check for the correctness and appropriatness of the methods. Please, add the dataset as a supplementary file.

**Response:** Thank you very much for your suggestions. We have added the dataset as Supplementary file S3.

**Comment 2:** The use of mixed effects models is sound. Linear mixed model could be an interesting option, but several aspects deserve more care. The model must fulfill some assumptions to ensure the reliability of the results. Those assumptions are completely overlooked. A complete analysis of the residuals must be included and tests (e.g log-likelihood ratio) should be included to check for the appropriateness of the random effects model. Moreover, some info on the random terms must be included (e.g. the variance of the random effects) and, as I am guessing that Gaussian distribution is taken from granted, if the assumptions on the random terms are also fulfilled.

**Response:** In the mixed effect models for repeated measures (MMRM), only correlation structure between outcome variables between time points is needed to be modeled explicitly [23]. Accordingly, the required assumption in this analysis is whether the correlation structure is correct. In this case, our analysis was unstructured; that is, we did not assume any structural assumptions for the correlation structure. MMRM is widely used for continuous outcome in longitudinal trials. We have described MMRM in the statistical analysis section (page 10, lines 153-155, 157-159).

[23] Mallinckrodt CH, Clark WS, David SR. Accounting for dropout bias using mixed-effects models. J Biopharm Stat. 2001;11: 9-21.

**Comment 3:** Please, provide inference on the baseline characteristics as well, bearing in mind that any parametric statistical test is based on some assumptions to be fulfilled.

**Response:** Statistical tests for baseline characteristics are not meaningful because these statistical strategies are not of interest in this trial, and a significance difference will occur with a probability of 0.05 [ref]. Therefore, we should interpret the difference based on a clinical viewpoint. In this study, there was no clinically meaningful difference in baseline characteristics between groups. In order to provide clarification, we have changed “The RHI value of the subjects was 0.61 ± 0.26 in the placebo group and 0.63 ± 0.29 in the high H_2_ group, and there was no significant difference.” to “Additionally, there was no significant difference in the RHI value of subjects between the groups (placebo group: 0.61±0.26, high H_2_ group: 0.63±0.29). We considered that the subjects in these two groups had physiologically similar characteristics in terms of endothelial function.” (page 11, lines 173-176).

[ref] Pocock SJ, Assmann SE, Enos LE, Kasten LE. Subgroup analysis, covariate adjustment and baseline comparisons in clinical trial reporting: current practice and problems. Stat Med. 2002;21: 2917-2130.

**Comment 4:** The way results are presented are somehow confusing to me. I would expect to see regression parameters, in which the placebo is taken as reference category. I do not really get how bot Placebo and Treatment can be included in a single model (as p-values are reported for both). Moreover, the term LS is not explained in the text. Please, report the results in a more common way, focusing on regression parameters and heterogeneity due to the longitudinal stracture in the data. Something should be said on the interaction effect as well.

**Response:** We have revised Tables 3-6 and summarized the data in Tables 2 and 3 for simplicity. The MMRM included the treatment group, time points, and interaction between the treatment group and the time points as fixed effects, with the RHI at baseline as a covariate. Therefore, we were able to estimate the covariate-adjusted mean and mean difference by estimated correlation coefficients. Additionally, the main purpose of this trial was to compare the adjusted mean between the groups, so we did not show many estimated parameters. LS is the covariate-adjusted mean, and we have revised the following sentence in the Results section to provide clarification (page 11, lines 173-176).

“Additionally, there was no significant difference in the RHI value of subjects between the groups (placebo group: 0.61±0.26, high H_2_ group: 0.63±0.29). We considered that the subjects in these two groups had physiologically similar characteristics in terms of endothelial function.”

Reviewer #3: The paper entitled “Peripheral endothelial function can be improved by daily consumption of water containing over 7ppm of dissolved hydrogen: A randomized controlled trial” describes the effect of oral intake of H2 improved the endothelial function assessed by reactive hyperemia peripheral arterial tonometry (RH-PAT).

In this study, subjects had an administration of 500mL of water every day. The subjects were divided into 2 groups by the property of water: high H2 water, and control. RH-PAT was performed at baseline, 1hour, 24 hours, and 14 days after the first intake. And the reactive hyperemia index (RHI), an index for a vascular endothelial function was compared between 2 groups. The authors found that the high H2 water group showed an increase in RHI in the later phase. And this effect was observed in subjects with lower RHI at baseline (lnRHI < 0.71). The authors have already reported this topic using flow mediated dilation (FMD), another index for endothelial dysfunction, and confirmed similar findings using another endothelial function test.
The paper suggests that oral intake of H2 might improve the endothelial function in healthy subjects having slightly reduced RHI. However, it is unclear if this treatment has a favorable effect on patients with impaired endothelial function.

Major comment
**Comment 1:** In general, the manuscript and too long and rambling. In Results, the same data were repeatedly shown in several figures and tables. This manuscript should be restyled with a more concise and clear way.

**Response:** Thank you very much for your suggestions. One of the factors that made the Discussion section of our manuscript too long is the limitation of the insight where the effects of H_2_ are originated from only the scavenger property against hydroxyl radical. Many researchers and clinicians in this field are pointing out the limitations of the scavenger theory. Our study’s results cannot be explained by the diminishment of hydroxyl radicals and/or peroxynitrite by H_2_.

Recently, we proposed new mechanistic insights of H_2_ aside from the scavenger theory, and the first evidence to explain the insights were accepted at the end of 2019 after we submitted this manuscript and published in February 2020 [35]. We have shortened the Discussion and restructured it to make it concise and clear by adding explanations for the mechanisms by which H_2_ can improve endothelial functions aside from the scavenger theory.

Further, we have deleted unnecessary sentences and revised text in the Introduction section.

Introduction: page 5, line 71 to page 6, line 78

“In some interventional studies, attempts to improve the reactive hyperemia index (RHI) without using a pharmaceutical approach, but instead focusing on exercise therapy and/or lifestyle improvement, have been reported in patients suffering from heart failure and metabolic syndrome. Improvements in RHI were also reported when postprandial hyperglycemia was treated pharmaceutically. Besides these disease conditions, it is important to identify safe and conventional ways for achieving relatively high (healthy) RHIs in the small vessels of healthy individuals.”

Deleted sentences

“Although the evidence regarding the use of RH-PAT is lacking, the results obtained and reported after using this relatively new method are similar to those obtained for FMD, and the convenience and/or reproducibility of the methodologies are more universal in RH-PAT compared to that in FMD

“This type of evaluation can elucidate the prognostic value of a new therapeutic modality, as well as determine the strategies for preventing any deleterious events associated with the use of this method.”

We have also restructured the Results section to shorten it and focus on the RHI values. We have revised Table 1 to clearly present the basic data of the subjects by combining the baseline data in Table 2, and we renumbered the tables. Data in Tables 2-5 include the same data of Figures 2 and 3, as you pointed out. We deleted the column for “Observed Value” from Tables 2-5. Although we considered whether Table 2-5 were essential, we considered that they are necessary for the statistical considerations that reviewer #2 requested. We have combined the data in Tables 2 and 3 into Table 2, and combined the data in Tables 4 and 5 into Table 3 in order to simplify the presentation for the statistical data.

We have deleted the following sentences from the Discussion section in order to shorten and restructure it.

“So far, we have discussed the potential that H_2_ can react and dissociate to donate electrons to the highly active radical molecules such as ^•^OH and ONOO^-^. According to guidelines on the safety of ingesting H_2_, therapeutic applications of H_2_ including clinical approaches have been emerging in the past decade.”

“Apart from the redox-related signaling including NO and the surrounding radical molecules such as H_2_O_2_, EDHF is attributed to electrochemical reactions by hyperpolarization of the endothelial cells first. The changes in the membrane potential of the endothelial cells can reach -85 ~ -80 mv, which is 10 ~ 30 mv below the basal membrane potential of arteries and arterioles. This hyperpolarization is dependent on the activation of both endothelial Ca^2+^-sensitive K^+^-channels, the small K_Ca_2.3 and K_Ca_3.1. The efflux of K^+^ affecting the contiguous smooth muscle cells connected to the gap junctions is the primary factor in the mechanism of vasodilation of relatively small arteries. Because H_2_ is a stable molecule requiring 436 kJ/mol to cleave the covalent bond, and this property simultaneously permits the safety of H_2_ in our body, it is unlikely that the chemical reactions of H_2_ are directly involved in the mechanisms of EDHF.”

“In this study, healthy meant that no medications were required and/or not likely to become ill. In this study, however, quite a few subjects had RHI values below 0.71 despite their thinking that they were healthy (22 subjects in the placebo group and 19 subjects in the high H_2_ group).”

“The RH-PAT in the small vessels may be influenced by volume loading more than by the diameter of the brachial artery as measured in FMD test. Furthermore, such loading of volume during the test may strain the sympathetic nerve and affect the PAT data. Within the high H_2_ group, an improvement was seen at 1 h after the drink in addition to at 24 h and 2 weeks after ingestion of the drink. This is consistent with the results we previously reported using FMD, where an H_2_-specific improvement was observed within 1 h after the consumption of 3.5 mg of H_2_. Currently, however, we could not assure the immediate efficacy of H_2_ on the peripheral artery, because of the presence of the placebo effect at 1 h in the low RHI group. Regarding the effect of H_2_ on RHI at 24 h, there was significant improvement from the RHI before consumption only within the H_2_ groups (Table 4), whereas in the low RHI groups, the difference from the value before ingestion was improved in the both of the placebo and H_2_ group. Further investigations about the immediate effects of a single ingestion of H_2_ within 24 h are necessary.”

Minor comments

Introduction
**Comment 2:** The introduction is too long, and describes too much for the comparison between FMD and EndoPAT.

**Response:** We have deleted sentences from the Introduction section to shorten and restructure it, as noted above in our response to your comment 1.

We have added the following sentences in the Discussion section (page 22, line 311 to page 23, line 334), according to the recent finding about the new properties of H_2_.

“It should be noted that measuring RH of the peripheral microcirculation in which occlusion of the brachial artery following the release of blood flow is known as the model of ischemia reperfusion (I/R) injury, although the function of the vasodilation of the microvasculature is not fully understood [29]. The mechanisms of the cellular damage by I/R injury are believed to be caused by the superoxide burst in the mitochondrial respiratory chain. Particularly, the reverse electron transport accompanied by the elevated mitochondrial membrane potential is the major factor of electron leakage in mitochondrial complex I [30]. Recently, we have reported that H_2_ reduces the mitochondrial membrane potential in the living cultured cells [31]. In the study, the overproduction of superoxide with the high ratio of NADH/NAD^+^, which reflects the highly reduced state of mitochondria during hypoxia following I/R injury [32], was suppressed by H_2_. This situation corresponds to the right circle in Figure 4. On the basis of these most recent mechanistic insights of H_2_ [31, 33], we suppose that H_2_ may improve endothelial function via the mitochondrial mechanisms in healthy subjects. H_2_ may shift the circle in Figure 4 from right to left by reducing the elevated mitochondrial membrane potential as well as the overproduction of superoxide under the condition with hypoxia presented in the right circle.

Thus far, the limitations to explain the mechanisms of the H_2_ effect on a wide range of diseases or pathological states based on the scavenging property of H_2_ with respect to highly reactive radicals have been discussed [34, 35]. In blood flow, the availability of H_2_ and/or the frequency with which it reacts with target radicals seems insufficient to explain the distinct biological effects of H_2_ in the peripheral vasculature. In the present study, the continuous scavenging of deleterious radicals by H_2_ is excluded by the single intake of high H_2_ water in a day. Therefore, we consider that the effects of H_2_ herein are occur via the mitochondrial mechanisms described in Figure 4 [30-33].”

**Comment 3:** In the introduction (line 85), the authors stated: “the nature of the rosy faces often observed….”. Do the authors have a reference for this?

**Response:** No, it is just a consensus among the clinicians treating H_2_; we do not have a reference to support this statement. We have deleted it.

Methods
**Comment 4:** In line 121, “both 2d fingers” should read “both index fingers”.

**Response:** Thank you very much for your advice. We have revised the text accordingly (page 7, line 105).

**Comment 5:** In this paper, RH-PAT was performed in the afternoon. The authors should describe the breakfast and other oral intakes like caffeine.

**Response:** We have described the 6 h fasting as follows (page 7, lines 95-96): “All subjects were asked to fast and avoid drinking caffeinated drinks or ingesting sugar for 6 h before the test.” We have also described the breakfast (page 7, line 96): “All subjects were asked to fast and avoid drinking caffeinated drinks or ingesting sugar as well as breakfast for 6 h before the test.”

**Comment 6:** The authors should describe the statistical analysis more precisely.

**Response:** We responded to the queries and requests of reviewer #2 about the statistical analysis, and we described MMRM (page 10, lines 153-155, 157-159).

Results
**Comment 7:** The authors divided subjects into low and high RHI groups with a cut-off of 0.71. They should explain how they conduced 0.71 as a cut-off.

**Response:** We explained the cut-off in the Discussion section, and we have revised the sentence and added a comment in the Results section.

Results: page 16, lines 215-217

“In order to identify the influence of the RHI value before ingestion on the data at each time point, we divided the subjects into two groups with the cut-off value of 0.71 (see Discussion section).”

Discussion: page 18, line 247 to page 19, line 259

“Although not intended for healthy subjects, there are several investigations on the prognostic value of RHI for predicting cardiovascular risks. The RHI values reported were 0.531 in 577 patients at high risk for cardiovascular events by Matsuzawa et al [6] and 0.4 in 270 low-risk outpatients with chest pain by Rubinshtein et al [7]. It has been also reported that the risk of arrhythmia recurrence could be estimated by an RHI value of 0.618 in 92 participants with atrial fibrillation undergoing catheter ablation [23]. Orthopedic patients who underwent total hip or knee arthroplasty and avoided postoperative deep vein thrombosis (DVT) had preoperative ln-RHI values of 0.71 ± 0.25 [24]. Among these reports, the border value of 0.71 for the orthopedic patients avoiding DVT seems to be close to that of the healthy masses. To predict future endothelial health in healthy subjects in this study, it seemed to be appropriate to set the threshold for RHI above the values for patients with cardiovascular risks. We set the RHI value at 0.71 in order to divide the subjects into two groups.”

Discussion

**Comment 8:** In line 295-297, the authors stated: “The RH-PAT in the small vessels may be influenced by volume loading…”. Please show some references.

**Response:** Thank you for pointing out this reference. Because the comments concerning the placebo effects were based on our speculative opinion, we searched for appropriate references. Unfortunately, we could not find a reference that clearly explained the relationship between hyperemia of peripheral circulation and intake of 500 ml of water within 1 h. Therefore, we deleted the explanation of the placebo effect attributing to overload of the volume on the peripheral vasculature in the condition of low RH. Instead, we stated (page 19, line 266 to page 20, line 268), “…although it is currently unclear whether it is merely the placebo effect or there is another factor that causes the improvement of RHI within 1 h after the placebo group drinks the water containing N_2_.”

**Comment 9:** In line 296-297, the authors said that loading of volume may strain the sympathetic nerve and affect PAT data. One of the advantages of RH-PAT is that RH-PAT might cancel the effect of systemic autonomic nervous tone by normalizing with contralateral PAT.

**Response:** Thank you very much for pointing this out. This comment is in contradiction with the theory of the PAT test so we deleted it. We also deleted the unnecessary sentence explaining the effects at 1 h compared with the results of the FMD test.

6. PLOS authors have the option to publish the peer review history of their article ([what does this mean?](https://journals.plos.org/plosone/s/editorial-and-peer-review-process#loc-peer-review-history)). If published, this will include your full peer review and any attached files.


If you choose “no”, your identity will remain anonymous but your review may still be made public.


**Do you want your identity to be public for this peer review?** For information about this choice, including consent withdrawal, please see our [Privacy Policy](https://www.plos.org/privacy-policy).

Reviewer #1: No

Reviewer #2: No

Reviewer #3: No


[NOTE: If reviewer comments were submitted as an attachment file, they will be attached to this email and accessible via the submission site. Please log into your account, locate the manuscript record, and check for the action link "View Attachments". If this link does not appear, there are no attachment files to be viewed.]


While revising your submission, please upload your figure files to the Preflight Analysis and Conversion Engine (PACE) digital diagnostic tool, <https://pacev2.apexcovantage.com/>. PACE helps ensure that figures meet PLOS requirements. To use PACE, you must first register as a user. Registration is free. Then, login and navigate to the UPLOAD tab, where you will find detailed instructions on how to use the tool. If you encounter any issues or have any questions when using PACE, please email us at [figures@plos.org](mailto:figures@plos.org). Please note that Supporting Information files do not need this step.
