# Supplementary material for: Peripheral endothelial function can be improved by daily consumption of water containing over 7 ppm of dissolved hydrogen: A randomized controlled trial
Source: PLoS One. 2020 May 29;15(5):e0233484. doi: 10.1371/journal.pone.0233484 (PMC7259729; doi:10.1371/journal.pone.0233484)
Supplement: Attachment — Submitted filename: Response to reviewers.docx [file pone.0233484.s006.docx]

**Peripheral endothelial function can be improved by daily consumption of water containing over 7 ppm of dissolved hydrogen: A randomized controlled trial (Manuscript: PONE-D-19-26280R1)**

**Response to Reviewer #1**

Thank you for reviewing our manuscript and for your insightful comments. We have responded to each of your comments below, and have revised the manuscript accordingly.

**Comment 1:** The present study lacks a real atherosclerotic group with endothelial function (e.g., hypertensive patients, diabetes mellitus, dyslipidemia, and cardiovascular disease).

**Response:** Thank you for your further suggestions.

In the former round of revision, we emphasized the aim and goal of the present study according to your previous suggestion in the Abstract section:

“In this study, we aimed to investigate the effect of H_2_ on RH-PAT of the small arteries of fingers in healthy people.”

…and in the Introduction section:

“It is important to not only estimate the function of the endothelium of both conduit arteries and peripheral arteries in healthy people using these non-invasive methodologies, but to also identify the factors or methodologies that can improve endothelial function and prevent endothelial dysfunction in healthy people without administering pharmaceutical drugs.”

“Besides these disease-conditions, it is important to identify safe and conventional ways for achieving relatively high (healthy) RHIs in the small vessels of healthy individuals.”

Furthermore, we added the comments in the Study limitations as follows (page 27, lines 342-347):

“As the aim of this study was to investigate the effect of H_2_ on RH-PAT intended for healthy subjects to assess the significance of daily consumption of H_2_ to prevent the endothelial dysfunction of small arteries, data for an atherosclerotic group with a low RHI value are lacking. It is important to examine the effect of H_2_ on RH-PAT of people having a high risk of hypertension, cardiovascular diseases, diabetes mellitus and dyslipidemia due to atherosclerosis. The next study should include participants with these characteristics.”

**Comment 2:** A number of studies in vitro reported that hydrogen directly works several types of cells through the various mechanisms. However, it is doubt that dissolved hydrogen in hydrogen water is taken in the vasculature and the cell after drinking hydrogen water in humans.

**Response:** As we explained in the Methods section (page 9, lines 138-140), it has been clarified and agreed in this field that H_2_ is transferred into the human body and the hydrogen is absorbed in vasculature and circulated from ingested water containing H_2._ (reference [22]).

Moreover, we added reference [23] and revised the sentence as follows (pages 9-10, lines 140-142):

“In cases where rats were administered 15 mL/kg of 0.8 mM H_2_ water by a catheter or mice drank approximately 200mL/kg of 0.2–0.4 mM H_2_ water per day, the concentration of H_2_ in their blood was 5–6 μM and about 2 μM, respectively [22, 23].”

**Response to Reviewer #2**

Thank you for reviewing our manuscript and for your insightful comments. We have responded to each of your comments below, and have revised the manuscript accordingly.

**Comment:** I appreciate most the reply given by the authors to my comments.

There are still some points deserving a better clarification.

Previously, I commented that "A complete analysis of the residuals must be included and tests (e.g log-likelihood ratio) should be included to check for the appropriateness of the random effects model. Moreover, some info on the random terms must be included (e.g. the variance of the random effects) and, as I am guessing that Gaussian distribution is taken from granted, if the assumptions on the random terms are also fulfilled.". The authors reply is not satisfactory. The unstructured specification mentioned in their reply refers to the variance-covariance matrix of the random effects. This is quite a flexible assumption and I am fine with it. The issue is that the random effects follow some distribution. Again I guess it is the Gaussian one, but this must be checked somehow, as departure from the Gaussian assumption may lead to biased estimates. Moreover, as the author replied, random effects modelling is widely used for continuous outcome in longitudinal trials. This is absolutely true. But continuous outcomes may be distributed according to hundreds of different distributions. Again, the most used one is the Gaussian, i.e. the error term in the linear predictor is assumed to follow a zero-mean Gaussian distribution. This is a crucial assumption of the modelling. If heavy tails are evident, i.e. if the error term follows a t distribution instead, regression parameters and all the statistical inference are biased. This is the reason why the analysis of residuals must be provided, because the linear mixed model is based on some assumptions. If those assumptions are not fulfilled, the results are unreliable. Citing a paper in the J. Bioph. Stat is not a good choice, as dozens of statistical books exist (just to mention one https://www.routledge.com/Longitudinal-Data-Analysis/Fitzmaurice-Davidian-Verbeke-Molenberghs/p/book/9781584886587) and discuss longitudinal data analysis, mentioning how to perform a proper data analysis.

**Response**: Thank you very much for your detailed advice. As you guessed, we assumed a Gaussian distribution for the error term in MMRM. I checked distribution of the residuals in the MMRM model for Table 2 and Table 3. Please see attached figure below. The distribution appears to be normal/ Gaussian (upper right) and the points approximately fall on the line in QQ-plot (lower left). In addition, we performed a log-likelihood ratio test in order to check the fit of the MMRM model to the data and its p-values were 0.0483 and 0.3597 for Table 2 and Table 3, respectively. The p-value of Table 3 (0.3597) is relatively large because of the small sample size. We think that the MMRM model is appropriate for the data. We have added a related sentence to the Statistical analysis section (pages 10-11, lines 157-159, and footnotes to Table 2 (page 17, line 215) and Table 3 (pages 20, line 237) as follows:

(Page 10-11, lines 157-159)

“Furthermore, we assumed a Gaussian error term in the model. We performed a log-likelihood ratio test and residual analysis in order to check the fit of the model.”

(Page 17, line 215)

“*p*-value of log-likelihood test: 0.0483”

(Page 20, line 237)

“*p*-value of log-likelihood test: 0.3597”

Furthermore, thank you for providing us with an appropriate reference. We replaced reference [24] according to your suggestion as follows:

[24] Garrett Fitzmaurice, Marie D, Geert V, Geert M. (2008). Longitudinal data analysis. Chapman and Hall/CRC.

Residual analysis for Table 1

Residual analysis for Table 2
